# Neurodegenerative Diseases: Molecular Mechanisms and Therapies 2nd Edition

**DOI:** 10.3390/ijms252111334

**Published:** 2024-10-22

**Authors:** Zhi Dong Zhou, Alexandre Hiroaki Kihara

**Affiliations:** 1 National Neuroscience Institute of Singapore, 11 Jalan Tan Tock Seng, Singapore 308433, Singapore; 2 Signature Research Program in Neuroscience and Behavioral Disorders, Duke-NUS Graduate Medical School Singapore, 8 College Road, Singapore 169857, Singapore; 3 Neurogenetics Laboratory, Universidade Federal do ABC, São Bernardo do Campo 09606-045, SP, Brazil; 4 Center for Mathematics, Computing and Cognition, Universidade Federal do ABC, São Bernardo do Campo 09606-045, SP, Brazil

Neurodegenerative disorders are multiple chronic neurological diseases that pose a serious public health risk to our society, especially among the aging population. The growing longevity and declining fertility rate raise the prevalence of neurodegenerative diseases, such as Alzheimer’s disease (AD), Parkinson’s disease (PD), and amyotrophic lateral sclerosis (ALS), exacerbating society’s economic burden significantly. Following the first edition, the second edition of this Special Issue continues to present the most recent advances in molecular pathogenesis and therapies relating to neurodegenerative disorders.

The global prevalence of AD dementia, prodromal AD, and preclinical AD cases has been estimated to be 32, 69, and 315 million, respectively. These conditions totaled 416 million human subjects across the AD spectrum, or 22% of all individuals aged over 50 years [1]. So far, various rodent models have been used for AD research, including an AD model induced by the intracerebroventricular injection of streptozotocin. In this Special Issue, Da Silva et al. first studied the proteomics profiles of prefrontal cortex and hippocampus neurons in the streptozotocin AD model via mass spectrometry analysis. Based on differentially expressed levels of proteins, the authors observed deregulated signaling pathways in the AD model, demonstrating the potential pathological features at early stages of late-onset AD (LOAD).

Furthermore, the hall of AD experimental models has been recently enriched with three-dimensional (3D) brain and retinal organoid models [2,3]. The 3D organoid models have multiple advantages to classic two-dimensional (2D) cell culture models. However, the 3D organoid models are generated from induced human pluripotent cells and a common criticism is that these models lose aging-related epigenetic markers. This kind of drawback has recently been overcome with a miRNA-mediated 3D organoid protocol from the neuronal reprogramming of LOAD patient fibroblasts [4]. In addition, the generated brain organoids encompass microglial cells [5]. In fact, inflammatory substances such as β-amyloid (Aβ) and oxysterols can activate microglial cells to change their shape and functions, leading to cytokine secretion and the increased expression of MHC class II molecules. A recent study by Kim et al. investigated how oxysterol treatment affected the expression of heat shock protein 60 (HSP60), a chaperonin protein, in human microglial cells. Proteomics analysis showed that oxysterols dramatically boosted microglial cell HSP60 expression. Interestingly, Aβ1–42 also increased HSP60 expression, highlighting the role of HSP60 as a potential microglial activator and therapeutic target for neuroinflammatory diseases, including AD.

The development and discovery of new medications to treat AD is extremely costly. Since 1995, private expenditures on clinical-stage AD research and drug development have been estimated to be USD 42.5 billion [6], revealing that alternative approaches are needed for future studies. In this regard, AI and machine learning models have been employed as novel approaches to contribute to anti-AD drug discovery [7]. In this Special Issue, Nwadiugwu et al. used distinct machine learning models, logistic regression (LogR), k-nearest neighbors (KNNs), random forest (RF), support vector machine (SVM), extreme gradient boosting (XGB), and neural networks (NNs) to predict active and inactive compounds that have the capacity to inhibit glycogen synthase kinase 3β (GSK-3β), a well-documented protein kinase with increased kinase activity related to tau hyperphosphorylation and neurofibrillary tangles in AD. It is worth mentioning that AD treatment strategies are initially focused on delaying or stopping dementia, the progressive deterioration of mental functions that affects activities of daily living. However, AD is also associated with an increased risk of comorbidities, including neuropsychiatric symptoms (NPSs), which encompass agitation, anxiety, and depression. In this Special Issue, Li et al. systemically reviewed current AD treatments and highlighted emerging pharmacological approaches to reduce cognitive deficits and AD-induced NPSs.

It is known that common mood disorders, psychosis, impulse control disorders, and NPSs are also frequently manifested in PD [8]. Although around 85% of sporadic PD cases are not related to family history, some pathological genetic factors are associated with PD, including mutations of *α-Synuclein* (*SNCA*), *Leucine-rich repeat kinase 2* (*LRRK2*), and *Parkin* (*PARK2*) genes. The paramount importance of mutations in the *Parkin* gene was first reported 25 years ago by Tohru Kitada, who identified it as a responsible factor for the onset of autosomal recessive juvenile Parkinsonism [9]. In this Special Issue, Kitada et al. reviewed the *Parkin* gene’s unresolved functions, pathological concerns, and future research trends in PD.

In PD treatment, dopamine-replenishing medication with dopamine D2/D3 receptor agonists causes decision-making difficulties. The neurological processes underlying these side effects are unknown, yet they support pathological gambling. In the current issue, Kubota et al. explore the effects of pramipexole, a D3 receptor agonist, on decision making in a PD mouse model. The authors discovered that the hyperactivation of external globus pallidus neurons impaired decision making in the PD mouse model, identifying a potential mechanism and therapeutic target for pathological gambling induced by D2/D3 receptor medication in PD.

ALS, also known as Lou Gehrig’s disease, is a neurodegenerative disorder that affects motor neurons controlling voluntary muscle movement. The economic expense of ALS is estimated to be more than USD 1 billion per year in the US alone [10]. Currently, the pathological causes of most ALS cases are unknown, and only around 10% of ALS cases are associated with pathological genetic factors. There is a pathological link between ALS and retroviral reverse transcriptase activity and human endogenous retrovirus (HERV) expression. In healthy individuals, HERVs affect immune responses and cell fate specification. However, infections and gene mutations can disrupt the process, resulting in uneven HERV expression, which may be linked to diseases including neurodegenerative disorders [11]. In this Special Issue, Moreno-Martinez et al. found that there were no differences in HERVK (human endogenous retrovirus-K, HML-2 subfamily) expression levels in peripheral blood mononuclear cells and postmortem brain samples from ALS patients. However, they did find significantly enhanced expression of specific HERVK copies (Chr3-3 and Chr3-5) with altered levels of inflammation markers, particularly in the brainstem, compared with healthy controls. The observations are consistent with the possible pathological significance of specific HERVK copies in ALS.
